# Commemorating a decade of service: Reflections on Nigeria’s deployment of 196 public health professionals to Liberia and Sierra Leone during the 2014–2015 Ebola crisis

**DOI:** 10.4102/jphia.v17i1.1545

**Published:** 2026-01-21

**Authors:** Womi-Eteng O. Eteng, Olayinka S. Ilesanmi, Chinasa U. Imo, Waheed A. Bakare, Nweyi A. Okoro, Ahmed T. Abubakar, Sunny Chuku, Amaka P. Onyiah, Chioma Dan-Nwafor, Uchenna P. Anebonam, Sikiru O. Badaru

**Affiliations:** 1 Africa Centres for Disease Control and Prevention, Addis Ababa, Ethiopia; 2 University of Chicago Crown Family School of Social Work, Policy and Practice, Chicago, United States of America; 3 Research and Data Solution, Abuja, Nigeria; 4 Nigeria Field Epidemiology Training Programme, Abuja, Nigeria; 5 Africa Centres for Disease Control and Prevention, Abuja, Nigeria; 6 Nigeria Centre for Disease Control and Prevention, Abuja, Nigeria

**Keywords:** Africa CDC, Ebola virus disease, Nigeria, ASEOWA, global health security, outbreak response

## Abstract

The West African Ebola virus disease (EVD) epidemic of 2013–2016 was the largest on record, severely affecting Guinea, Liberia, and Sierra Leone, and overwhelming public health systems. Following Nigeria’s successful containment of its domestic EVD outbreak in 2014, the African Union Support to Ebola Outbreak in West Africa (ASEOWA) mission deployed 196 Nigerian public health professionals, the largest single-nation contingent, to Liberia and Sierra Leone. This commentary reflects on that deployment, highlighting operational contributions, innovations, and the enduring impact of the mission on Africa’s public health security landscape. The Nigerian team strengthened Ebola Treatment Units (ETUs), surveillance, epidemiological investigations, laboratory testing, infection prevention and control (IPC), community mobilisation, and restoration of essential health services. Significantly, no responder infections occurred under Nigeria’s deployment. The mission reinforced African-led outbreak response and contributed to the evolution of regional security structures, including the Africa Centres for Disease Control and Prevention (Africa CDC) and the African Union Volunteer Health Corps (AVOHC). A decade later, the deployment remains instrumental in shaping sustained public health workforce investments and integrated emergency preparedness systems across Africa.

## Introduction

The 2013–2016 West African Ebola virus disease (EVD) epidemic led to more than 28 500 cases and 11 000 deaths, marking the most consequential Ebola outbreak in history and exposing profound continental and global vulnerabilities in epidemic preparedness. Guinea, Liberia, and Sierra Leone were disproportionately affected, owing to fragile health systems, insufficient laboratory and surveillance capacity, and public mistrust of government health services. Overwhelmed health systems struggled to provide routine care, while social disruption, economic instability, and mortality among frontline health workers intensified the crisis.^1,2,3^

Nigeria successfully halted its 2014 EVD outbreak (July 2014 – September 2014) after only 20 cases and eight deaths, an achievement widely credited to robust incident management, urgent political commitment, integrated surveillance, extensive contact-tracing, and rapid deployment of digital field tools.^1,4,5^ Leveraging this experience, the African Union (AU) mobilised Nigeria to support regional response efforts. On 08 August 2014, the World Health Organization (WHO) declared the EVD epidemic a Public Health Emergency of International Concern (PHEIC), prompting the AU to establish the African Union Support to Ebola Outbreak in West Africa (ASEOWA) mission, the first AU-led continental health emergency mobilisation.^6,7^

Nigeria’s deployment of 196 public health professionals to Liberia and Sierra Leone under ASEOWA in December 2014 constituted the largest contribution from a single country. The contingent was the first country seconded and the largest single-nation deployment, underscoring Nigeria’s long-standing dedication to regional stability.^8^ The Nigerian contingent represented more than 25% of the 720 health workers mobilised by the AU and was instrumental in strengthening case management, surveillance, laboratory systems, infection prevention and control (IPC), and cross-border operations. The mission provided an important model of Africa-led emergency health workforce mobilisation and informed the eventual establishment of the Africa Centres for Disease Control and Prevention (Africa CDC), inaugurated in January 2017.^8,9^

This commentary reflects on the Nigeria ASEOWA mission, summarising the deployment strategy, key public health contributions, operational challenges, lessons learned, and the broader implications for health security on the continent.

## The birth of African Union Support to Ebola Outbreak in West Africa: An African-led response

**FIGURE 1:**
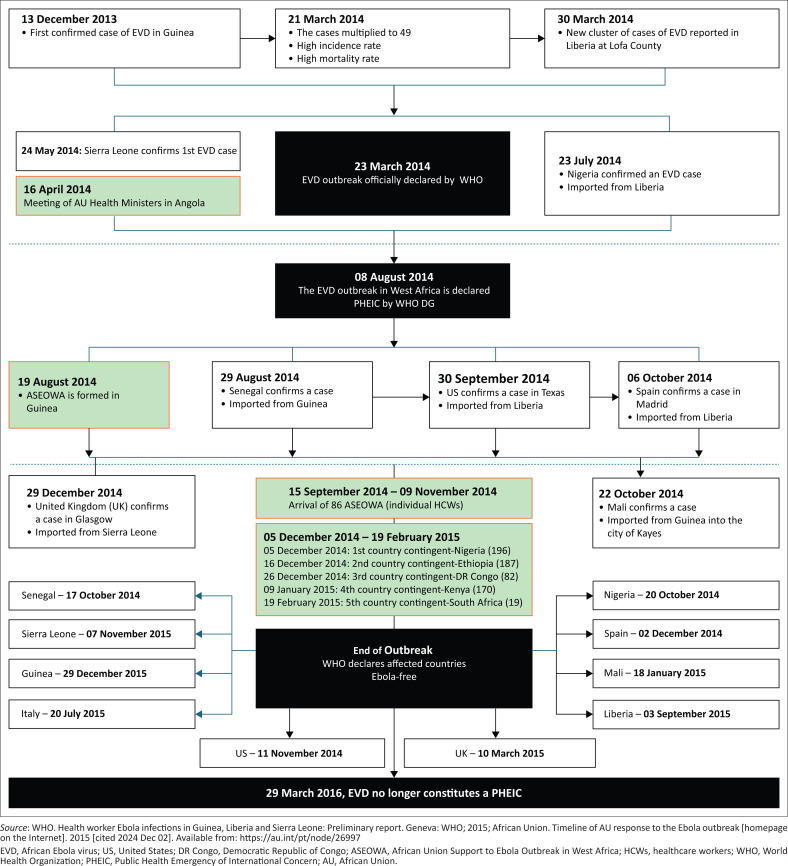
The 2013–2016 Ebola virus disease outbreak schema.

The outbreak was declared by the WHO on 23 March 2014, but by August of that year, ongoing transmission, rising mortality, and cross-border spread prompted escalating concern.^10^ In April 2014, the WHO collaborated with African Ministers of Health to accelerate response support. By August 2014, the 450th AU Peace and Security Council authorised the immediate deployment of civilian and military health assets. This political action led to the creation of a structured multicountry intervention reinforcing outbreak control and supporting health systems recovery in Liberia, Sierra Leone, and Guinea.^11^ Figure 1^3,6^ shows the timeline of the 2013–2016 Ebola outbreak declarations in various countries and deployment of responders.

African Union Support to Ebola Outbreak in West Africa featured two types of responders:

- *individual volunteers* recruited across AU member states
- *seconded personnel* deployed in-country under national arrangements.

Other AU-member country contributions included Ethiopia (*n* = 184), Kenya (*n* = 165), Democratic Republic (DR) of the Congo (*n* = 82), and South Africa (*n* = 19).^11^ An additional 115 health workers came from the Economic Community of West African States (ECOWAS), reinforcing the mission’s pan-African identity.

**FIGURE 2:**
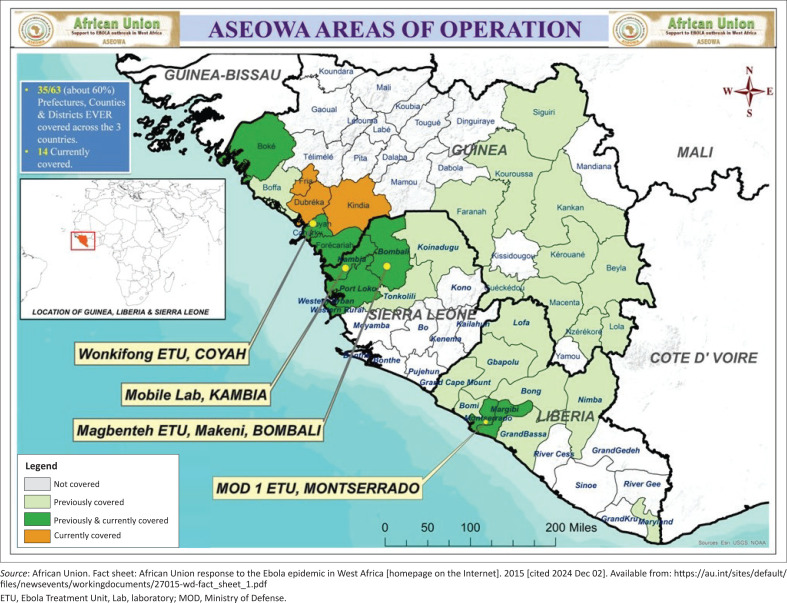
ASEOWA presence and operations across the three most affected countries.

The ASEOWA Concept of Operations (CONOPs) adopted the principle ‘one goal, one strategy, one operational plan’. It emphasised deployment integration into each host country’s National Ebola Response Centre to avoid duplicated functions and ensure operations aligned with national priorities. Skill profiles ranged from field epidemiologists, medical doctors, nurses, laboratory scientists, logisticians, data managers, IPC specialists, psychosocial teams, and burial units (Figure 2).^11^

In addition to EVD control, ASEOWA engaged in community-based interventions, clinical care restoration, and health system recovery.^12^ Many responders, including Nigerian personnel, later transitioned into national or multilateral leadership roles, reinforcing the mission’s long-term capacity-building influence.

## Nigeria’s deployment experience, deployment structure and composition

Nigeria’s contingent was 196 public health professionals; 58% males, and the ages ranged from 24 years to 58 years (mean age: 37.1 years). Responders’ assignments to duties were guided by epidemiological needs at the time of arrival: 57% were stationed in Sierra Leone, and the remainder in Liberia. The group operated across 10 of Liberia’s 15 counties and seven of Sierra Leone’s 14 districts.

The Nigeria Centre for Disease Control (NCDC), which had recently coordinated Nigeria’s domestic containment, provided technical leadership. Deployment rapidly followed training and predeparture orientation supervised by the AU. Personnel were positioned within national Emergency Operations Centre (EOC) structures, Ebola Treatment Units (ETUs), district surveillance units, and laboratory networks.

## Core operational roles and contributions

The Nigerian team carried out essential public health functions across eight domains:

- **Case management in ETUs**
  Nigerian clinicians provided triage, clinical care, supportive management, oxygen therapy, and discharge monitoring. They strengthened operations at major ETUs, including 100-bed treatment centres in Monrovia (Liberia) and Bombali (Sierra Leone).
- **Surveillance and epidemiology**
  Epidemiologists bolstered case investigation, verification of EVD deaths, active case finding, and data triangulation. Their deployment strengthened district-level surveillance continuity, especially during peak transmission periods.
- **Contact identification and follow-up**
  Working with local response teams, they supported enumeration of contacts, monitoring of symptoms during the 21-day follow-up period, and field validation of suspected chains of transmission.
- **Laboratory systems strengthening**
  Laboratory scientists supported specimen reception, handling, and confirmatory testing. A Nigerian-supported mobile laboratory, initially established for Lassa fever research, became a pivotal diagnostic asset in Sierra Leone, reducing delays caused by transporting samples over long distances.^7^
- **Infection prevention and control**
  IPC training, facility assessments, clinical mentoring, and proper use of personal protective equipment (PPE) were integral components of the response. The Nigerian contingent recorded zero infections among its responders, demonstrating adherence to IPC protocols and effective predeployment training.
- **Hospital support and restoration of essential services**
  As the outbreak peaked, routine services were halted in many areas. Nigerian volunteers assisted in emergency surgery, antimicrobial stewardship, maternal and child health services, obstetric interventions, and triage systems. Across Liberia and Sierra Leone, 93 hospitals and health facilities received direct or indirect clinical and IPC support, helping restore care delivery capacity.
- **Data management and information systems**
  Volunteers worked alongside national response centres to ensure timely data reporting, entry, validation, and analytics. Nigeria Centre for Disease Control experience using mobile-based data tools provided critical value; Nigeria’s innovative field digital platforms (ODK or FormHub) were conceptual models for the later development of Surveillance, Outbreak Response Management and Analysis System (SORMAS), now a widely used global digital platform.^13^
- **Community engagement and psychosocial support**
  The Teams collaborated with community structures, survivors, and social mobilisers to address stigmatisation and facilitate safe burial practices. Support for national risk communication efforts helped rebuild trust and counter misinformation.

Through these roles, the Nigerian contingent reinforced locally driven outbreak management while enabling the gradual restoration of essential health services.

## Operational challenges

Despite the cohort’s successful deployment, Nigeria’s ASEOWA teams encountered multifactorial operational and contextual challenges that shaped field implementation and response efficiency.

### Severe workforce shortages

Liberia and Sierra Leone experienced catastrophic health worker attrition because of EVD-related mortality, infections, and burnout.^14,15^ Staffing deficits amplified clinical demands on ASEOWA teams, necessitating longer shifts, intensified IPC vigilance, and broader clinical roles beyond Ebola-specific care. Strict adherence to IPC protocols ensured zero infections among the Nigerian cohort, reinforcing the value of strong clinical governance systems.^12^

### Supply chain limitations

The scarcity of PPE, laboratory consumables, disinfectants, and pharmaceuticals impeded effective case management and IPC implementation. Remote health facilities reported irregular delivery of supplies and delayed sample transport. African Union Support to Ebola Outbreak in West Africa addressed some of these challenges through temporary on-site reprocessing systems and improved triage protocols; however, structural supply deficits remained persistent barriers.

### Geographic inaccessibility and poor infrastructure

The poor road network, especially during the rainy season, affected epidemiologic surveillance and contact-tracing operations. Mountainous terrain in the districts of Sierra Leone and Liberia further complicated movement. Aerial and water transport were rarely available for health teams. Consequently, delays in case reporting, sample transportation, and emergency referrals were common.

### Community mistrust and social resistance

Deep mistrust in formal health systems, reliance on traditional healers, and misinformation about Ebola transmission complicated containment. Many families resisted case reporting and safe burial procedures because of cultural norms. Nigerian responders working with national risk communication teams leveraged local leaders, Ebola survivors, and community health volunteers to promote infection prevention messages and safe care-seeking.

### Psychosocial burden on responders

Psychological stress was significant because of prolonged exposure to severely ill patients, high mortality, and fear of contagion. Nigerian psychosocial teams provided peer support and developed coping protocols informed by global mental health standards for humanitarian response.

### Data fragmentation and limited information communication and technology infrastructure

At the time, most surveillance relied on paper-based reporting, limiting timeliness and accuracy. Partnerships between ASEOWA teams and national surveillance units partially mitigated this through data harmonisation, but information communication and technology (ICT) limitations constrained near-real-time epidemic intelligence. These challenges later informed stronger digital surveillance design, including SORMAS and other platforms.^13^

## Reflections and lessons learned

The Nigeria ASEOWA deployment yielded valuable insights central to the regional health security discourse. Key reflections include:

### African solidarity enhances regional preparedness

The deployment demonstrated the feasibility and effectiveness of intracontinental health workforce mobilisation for epidemic response. Nigeria’s deployment capacity, rooted in prior domestic outbreak experience, created an opportunity to transfer context-appropriate interventions to neighbouring nations, countering the narrative that Africa must rely primarily on external partners during crises.

### Prepared workforces enable rapid health security gains

Critical response capacities, surveillance, ETU case management, and IPC relied on skilled personnel. Predeployment training ensured responders possessed the necessary competencies. This informed later operationalisation of the African Volunteer Health Corps (AVOHC), now a structure for continental emergency workforce readiness.^16^

### Integration with national EOCs improves operational synergy

Nigeria’s alignment with existing national response coordination mechanisms reduced duplication, accelerated decision-making, and strengthened operational cohesion. This integrated coordination supports the current Africa CDC’s operational doctrine on emergency deployments.

### Sustaining essential health services must be prioritised

Healthcare systems in Liberia and Sierra Leone faced dramatic service reductions.^12^ The Nigerian contingent’s clinical reinforcement allowed gradual restoration of routine care, including emergency surgeries, obstetric services, and inpatient care. Maintaining essential services is now part of the global outbreak-response doctrine endorsed by the WHO.

### Digital surveillance innovations matter

The domestic EVD response in Nigeria pioneered the use of FormHub and ODK-based mobile tools for digital contact-tracing. Lessons from these systems eventually shaped the development of SORMAS, now deployed across West Africa and used for coronavirus disease 2019 (COVID-19), mpox, and other outbreaks.^13^

### Community engagement determines outbreak trajectory

Engagement of traditional authorities and survivors helped to improve outbreak literacy, reduce stigma, and accelerate reporting. Today, risk communication and community engagement (RCCE) is a core component of the WHO and Africa CDC response, demonstrating the enduring value of this lesson.

## Broader implications for regional health security

The ASEOWA deployment galvanised momentum towards building Africa’s permanent epidemic-preparedness infrastructure. Key long-term implications include:

### Foundation for Africa Centre for Disease Control

Lessons from ASEOWA and Ebola accelerated the establishment of Africa CDC (established in 2017), which now leads continent-wide emergency preparedness, workforce training, and coordinated response.^17^ The Nigerian contingent’s work informed operational models for rapid response team (RRT) deployment, laboratory network coordination, and IPC strengthening.

### Establishment of African Volunteer Health Corps

The AU Volunteer Health Corps emerged to institutionalise emergency public health workforce systems. The AVOHC has since supported multiple outbreak responses, including mpox, COVID-19, cholera, Ebola, and other humanitarian health emergencies.

### Strengthening regional surveillance and data systems

The outbreak exposed gaps in early detection and cross-border surveillance. Innovations such as SORMAS, national Public Health Emergency Operations Centres (PHEOCs), and the ECOWAS Regional Centre for Surveillance and Disease Control (RCSDC) were strengthened.^7,18^

### Expansion of infection prevention and control infrastructure and training

Zero responder infections within the Nigerian cohort reinforced the importance of IPC protocols. Many training curricula developed during ASEOWA now inform the Africa CDC and national IPC programmes.

### Workforce retention and leadership development

Several Nigerian responders assumed leadership positions in Nigeria and internationally, contributing to a stronger continental talent pipeline. This reflects the catalytic role of outbreak deployments in advancing public health leadership.

## Policy recommendations

To consolidate gains and prepare for future health crises, the following policy actions are proposed:

- Anchor AVOHC within national strategic plans to ensure predictable mobilisation and accountability.
- Increase domestic financing for workforce training, digital surveillance, and laboratory capacity.
- Institutionalise continuous simulation exercises and cross-border RRT drills to strengthen preparedness.
- Expand community health networks and survivor-engagement programmes.
- Integrate outbreak-response surge staffing plans within national PHEOCs.
- Strengthen emergency logistics systems to reduce.

## Conclusion

A decade after the largest EVD outbreak in history, Africa continues to face complex infectious disease threats, including COVID-19, cholera, mpox, and recurrent zoonoses. The Nigeria ASEOWA deployment demonstrated the value of continental solidarity, skilled emergency workforce capacity, and integration with national response systems. The mission accelerated the development of critical institutional architecture, including Africa CDC, AVOHC, and regional surveillance networks and provided important lessons about IPC, health-service continuity, digital surveillance, and RCCE.

Strategic investments made following this mission must now be expanded and sustained. These include strengthening preservice public health workforce development, streamlining rapid deployment frameworks, and strengthening PHEOC and digital systems. Future progress will require governments, multilateral partners, and civil society to maintain momentum and support long-term systems development.

Nigeria’s role in the 2014–2015 EVD response reflects the possibility and power of African-led solutions. Sustaining these gains will enable the continent to respond more effectively to future epidemics and help ensure global health security.
